# Isolation and expression analysis of *CsCML* genes in response to abiotic stresses in the tea plant (*Camellia sinensis*)

**DOI:** 10.1038/s41598-019-44681-7

**Published:** 2019-06-03

**Authors:** Qingping Ma, Qiongqiong Zhou, Canmei Chen, Qiaoyun Cui, Yuxin Zhao, Kun Wang, Emmanuel Arkorful, Xuan Chen, Kang Sun, Xinghui Li

**Affiliations:** Tea Research Institute, Nanjing Agricultural University, No. 1 Weigang avenue, Nanjing, 210095 China

**Keywords:** Transcriptional regulatory elements, Reverse transcription polymerase chain reaction

## Abstract

Calmodulin-like (CML) proteins are a class of important Ca^2+^ sensors in plants, which play vital roles in regulating plant growth and development and response to abiotic stress. Tea plant (*Camellia sinensis* L.) is the most popular non-alcoholic economic beverage crop around the world. However, the potential functions of CMLs in either tea plants growth or in the response to environmental stresses are still unclear. In the present study, five *CsCML* genes (*CsCML*16, *CsCML*18-1, *CsCML*18-2, *CsCML*38, and *CsCML*42) were isolated from tea plant, and functionally characterized. The *CsCML* genes showed diverse expression patterns in leaves, roots, old stems, immature stems and flowers of tea plants. To investigate the expression changes of the genes under various abiotic stresses and ABA treatment, time-course experiments were also performed, the results indicated that the expression levels of *CsCML16*, *18-2* and 42 were significantly induced under low temperature and salt condition, while *CsCML38* was induced distinctly under drought stress and ABA treatment. Overall, Cs*CML* genes showed diverse function in tea plant under various stimuli. These results will increase our knowledge of the significance of *CsCML* genes in tea plant in response to abiotic stresses and hormone treatments.

## Introduction

Plant growth and development is negatively affected by various abiotic and biotic stresses including low temperatures, salinity, drought, heavy metal toxicity, pathogens and insect attacks^1^. These stresses impede plant’s growth, survival, geographical distribution and agricultural productivity across the world. Plants undergo multiple physiological and biochemical responses to survive, and these responses depend on different gene expressions to combat and overcome stresses by cross talk of stress signals under different stress conditions^2^. Signal transduction is accomplished predominantly through a Ca^2+^-induced conformational change. The Ca^2+^ signals are perceived by various Ca^2+^ binding proteins or Ca^2+^ sensors, including calcineurin-B-like (CBL) proteins, calmodulin (CaM), CaM-like (CML) proteins and calcium-dependent protein kinases (CDPKs). These proteins usually include EF-hand motifs with helix-loop-helix (HLH) structure^3^. The EF-hand motif is reported as a conserved domain identified in Ca^2+^ binding proteins that are responsible for cooperative binding with Ca^2+^ ion^4^. After binding, the Ca^2+^ sensor proteins induce a molecule structural change and interact with their downstream target proteins, which couple with changes in Ca^2+^ concentration to regulate biochemical activities in cells^5,6^.

Plants possess a large family of unique CML proteins that contain the EF hand structure, which is similar to that in CaM^7^. CMLs contain 1–6 EF-hand motifs, but do not possess other known functional domains. A number of CMLs have been found in many plant species, such as *Arabidopsis*^8^, rice^9^, tomato^10^ and Chinese cabbage^11^. The identified CML proteins have been shown to respond to different stresses and hormone stimuli^12–14^. In *Arabidopsis*, the expression levels of *AtCML8* gene were significantly induced by salinity and salicylic acid (SA)^15^. Additonally, knockout of *AtCML9* improved the tolerance of *Arabidopsis* under salinity and drought treatments by increasing the contents of amino acids^16^. In rice, *OsCML4* could improve drought tolerance through effectively scavenging of reactive oxygen species (ROS)^17^. Over *ShCML44* was variously expressed in all wild tomato tissues and was intensely up-regulated by cold, drought, salinity and plant hormones treatments^18^. The aforementioned evidence indicated that CML proteins have critical roles in Ca^2+^ signal transduction in plant development and adaptation to abiotic stresses.

Tea plant (*Camellia sinensis* L.) is an important economic crop, which provides leaves for producing non-alcoholic beverage^19^. As a leaf-harvested crop, tea plant is inevitably confronted with various adverse environment stresses throughout the whole life cycle, such as drought^20^, salt^21^ and cold^22^ stresses, which seriously hinders the development of the tea industry. Although CMLs have been isolated and identified in many plant species, CML-mediated abiotic stress adaptation in tea plant has not been previously investigated, even though it is important to tea growth and production. In this study, in order to identify and characterize *CML* genes in tea plant, we identified five *CsCML* genes, and analyzed the gene expression patterns under abiotic stresses conditions and ABA treatment. The results of this study would be helpful to further characterize the abiotic stress responses in tea plants, and the breeding work of new tea germplasm resources with improved stress resistance.

## Results

### Cloning and sequence analysis of *CsCML* genes

**Figure 1:**
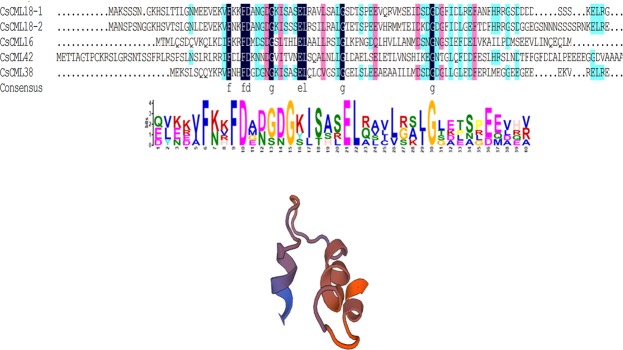
Conserved motifs of *CsCML* proteins. Alignment of the deduced amino acid sequences of *CsCML* proteins. (**A**) The sequence logo of the conserved CML domain including the Ca^2+^ binding EF-hand (a helix-loop-helix structure) motif was determined by MEME using amino acid sequences of *CsCML*. (**B**) Structure of the EF-hand domain.

A total of ten expressed sequence tags (ESTs) of *CsCMLs* were found from the transcriptome data (SRA accession number: SRR5075641). After removing the sequences with length shorter than 200 bp and splice error, five *CsCMLs* were finally obtained. They encoded five proteins with 136–201 amino acids, molecular weight of 16.06–22.48 KDa and isoelectric point of 4.14–4.72 (Table 1). All *CsCMLs* except *CsCML18-1* mapped to genes in the tea plant genome (www.plantkingdomgdb.com/tea_tree/)^23^, and no additional *CsCML* members were identified. With reference to the sequence similarity with homologous genes from other plant species, the five *CsCMLs* were named as *CsCML16*, *CsCML18-1*, *CsCML18-2*, *CsCML38* and *CsCML42*. *CsCML18-1* has one EF-hand conserved domain, while other four *CsCMLs* have two EF-hand conserved domains, indicating structural differentiation among *CsCML* genes (Fig. 1).

**Table 1:** Basic information of *CsCML* genes identified in tea plant.

Name	Gene ID in tea tree genome	Gene ID in transcriptome (SRR5075641)	Number of amino acids	Mw	Number of EF-hands	pI
*CsCML16*	CSA034174	CL6709.Contig1_All	136	17.58KDa	2	4.14
*CsCML18-1*	/	CL11681.Contig2_All	157	17.36KDa	1	4.55
*CsCML18-2*	CSA028811	CL11681.Contig1_All	166	18.31KDa	2	4.72
*CsCML38*	CSA025825	Unigene22722_All	144	16.06 KDa	2	4.27
*CsCML42*	CSA002945	Unigene16517_Al	201	22.48KDa	2	4.29

### Multiple sequence alignment and phylogenetic analysis

**Figure 2:**
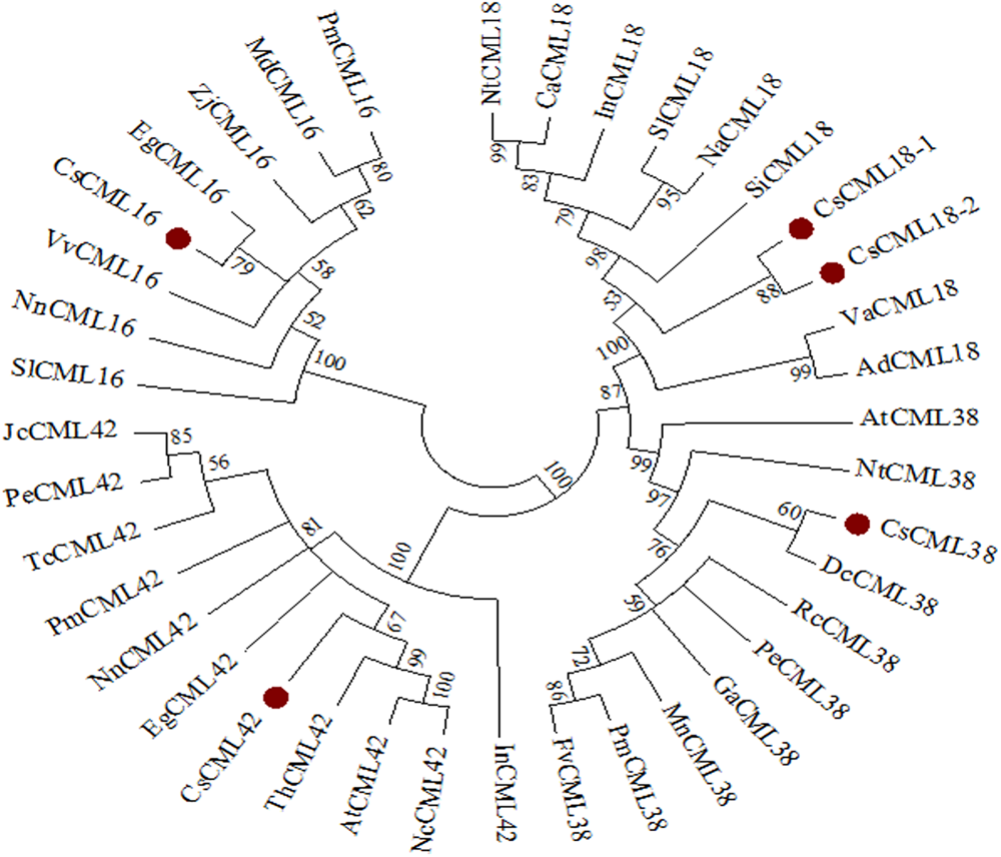
Phylogenetic relationship of *Cs*CML proteins with other plant species. *Camellia sinensis* (*Cs*CML16, *Cs*CML18-1, *Cs*CML18-2, *Cs*CML38, *Cs*CML42), *Arachis duranensis* (*Ad*CML18, XP_015931565.1), *Arabidopsis thaliana* (*At*CML38, OAP19420.1; *At*CML42, NP_193810.1), *Capsicum annuum* (*Ca*CML18, XP_016560844.1), *Daucus carota* subsp. Sativus (*Dc*CML38, XP_017246479.1), *Erythranthe guttate* (*Eg*CML16, XP_012855227.1; *Eg*CML42, XP_010036034.1), *Fragaria vesca* subsp. vesca (*Fv*CML38, XP_011467704.1), *Gossypium arboretum* (*Ga*CML38, XP_017621889.1), *Ipomoea nil* (*In*CML18, XP_019173083.1; *In*CML42, XP_019186611.1), *Jatropha curcas* (*Jc*CML42, XP_012090979.1), *Malus domestica* (*Md*CML16, XP_008339727.1), *Morus notabilis* (*Mn*CML38, XP_010111596.1), *Nicotiana attenuata* (*Na*CML18, XP_019257947.1), *Noccaea caerulescens* (*Nc*CML42, JAU17134.1), *Nicotiana tomentosiformis* (*Nt*CML18, XP_009628802.1; *Nt*CML38, XP_009596232.2), *Nelumbo nucifera* (*Nn*CML16, XP_010262279.1; *Nn*CML42, XP_010244597.1), *Populus euphratica* (*Pe*CML38, XP_011003944.1; *Pe*CML42, XP_011018929.1), *Prunus mume* (*Pm*CML16, XP_008243136.1; *Pm*CML38, XP_008231145.1; *Pm*CML42, XP_008244386.1), *Ricinus communis* (*Rc*CML38, XP_002509541.1), *Solanum lycopersicum* (*Sl*CML16, XP_004237205.1; *Sl*CML18, XP_004233476.1), *Sesamum indicum* (*Si*CML18, XP_011099473.1), *Theobroma cacao* (*Tc*CML42, EOY04379.1), *Tarenaya hassleriana* (*Th*CML42, XP_010537462.1), *Vigna angularis* (*Va*CML18, XP_017415815.1), *Vitis vinifera* (*Vv*CML16, XP_003631231.1), *Ziziphus jujube* (*Zj*CML16, XP_015895735.1).

The deduced amino acid sequences of five *CsCML* were analyzed by multiple sequence alignments. As shown in Fig. 2, *Cs*CML16 and *Eg*CML16 (*Erythranthe guttate*) clustered in the same branch and showed the highest similarity, followed by *Zj*CML16 (*Ziziphus jujube*), *Pm*CML16 (*Prunus mume*), *Md*CML16 (*Malus domestica*) and *Vv*CML16 (*Vitis vinifera*)*. Cs*CML18-1 and *Cs*CML18-2 were classified into the same cluster, indicating that they were paralogous genes. Both *Cs*CML18-1 and *Cs*CML18-2 showed high similarity to CML18 from other plant species. *Cs*CML38 clustered with *Dc*CML38 (*Daucus carota subsp. Sativus*), followed by *Rc*CML38 (*Ricinus communis*), *Pe*CML38 (*Populus euphratica*) and *Ga*CML38 (*Gossypium arboretum*). For *Cs*CML42, this gene shares highly similarity with *Th*CML42 (*Tarenaya hassleriana*), *At*CML42 and *Nc*CML42 (*Noccaea caerulescens*). Phylogenetic tree analysis revealed that the *Cs*CMLs amino acid sequences showed highly homologous to those of other plant species.

### Expression analysis of *CsCMLs* genes in different tissues

**Figure 3:**
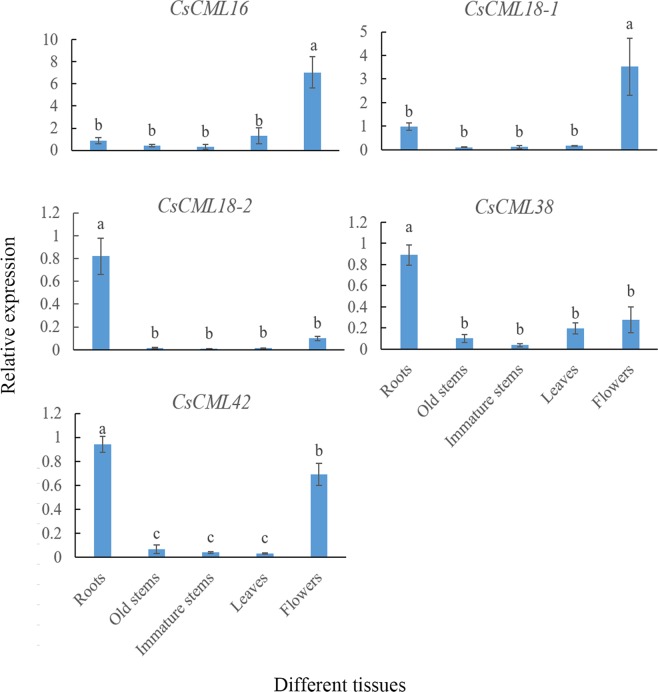
Analysis of tissue-specific expression pattern of *CsCML* genes in tea plant. Group comparison was performed with root as control. Different small letters mean significant differences (*P* < 0.05).

Tissue-specific gene expression profile might be associated with the specific physiological and developmental functions in plants. The expression levels of *CsCML* family gene in different tissues, including leaves, roots, old stems, immature stems and flowers, were examined by qRT-PCR. As shown in Fig. 3, all *CsCMLs* were expressed in different tissues of tea plant with the expression level of tea roots as control. The expression levels of *CsCML16* and *CsCML18-1* were remarkably higher in flowers than in other tissues. *CsCML16* also had a higher expression level in leaves than as expressed by other genes. However, their expression levels were induced slightly in other tissues, indicating that *CsCMLs* display tissue-specific expression in tea plant. The expression levels of *CsCMLs* in different tissues suggest that *CsCML* genes may have different functional variation in tea plant and this remains to be further investigated.

### Expression analysis of *CsCMLs* genes in tea plant under various abiotic stresses

**Figure 4:**
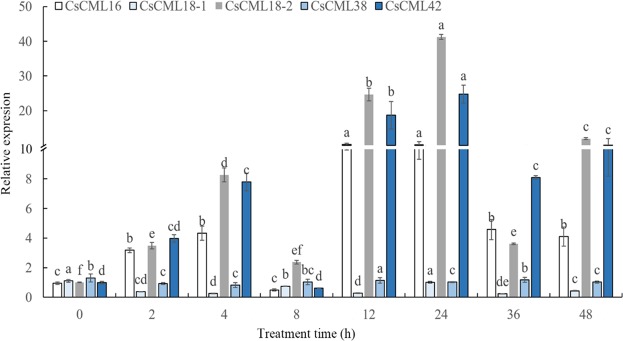
Expression profiles of *CsCML* genes in response to cold stress. Group comparison was performed with 0 h as control. Different small letters mean significant differences in *CsCML* genes at different time after cold stress (*P* < 0.05).

To explore the expression profiles of the *CsCMLs* genes under various abiotic stresses, time-course experiments of 5 *CsCML* genes in two-year-old seedlings treated with low temperature (10 °C), drought (PEG 6000), salinity (NaCl), and hormone (ABA) we performed. Generally, the 5 *CsCML* genes displayed distinctively expression patterns at different time points after stress treatments (Fig. 4). The expression levels of *CsCML16*, *CsCML18-2* and *CsCML42* were induced significantly by cold stress, and these genes showed highest expression levels at 24 h after cold stress. In contrast, the expression level of *CsCML18-1* was suppressed by cold stress. The reverse expression patterns between *CsCML18-1* and *CsCML18-2* revealed a diverse function in paralogs of *CML* genes in plants. In addition, *CsCML38* showed relative stable expressions under cold stress, which indicate that *CsCML38* is insensitive to cold stress.

**Figure 5:**
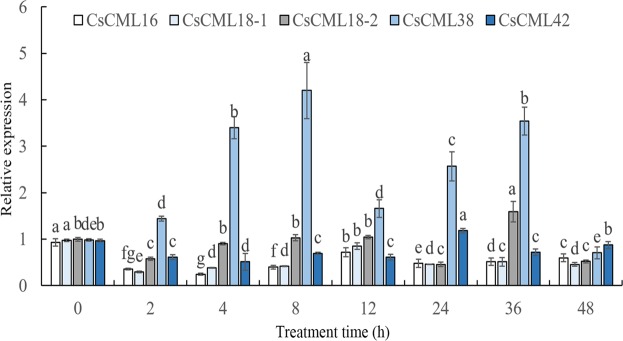
Expression profiles of *CsCML* genes in response to drought stress. Group comparison was performed with 0 h as control. Different small letters represent significant difference (*P* < 0.05).

Under drought stress caused by 20% PEG 6000 treatment, the expression of *CsCML38* was induced distinctly. However, expressions of *CsCML16* and *CsCML18-1* were reduced significantly after drought stress. The expression level of *CsCML18-2* had a little fluctuation between 0–12 h, but decreased significantly at 24 h, and then showed highest expression levels at 36 h. *CsCML42* was obviously inhibited at 4 h, followed by an increase until 24 h, and a decline at 36 h. The results indicate that *CsCML* genes showed different expression patterns under drought stress. In addition, the expression patterns of *CsCMLs* under drought stress were significantly different from that under cold and salt stress, suggesting that different response mechanisms were activated under different abiotic stresses (Fig. 5).

**Figure 6:**
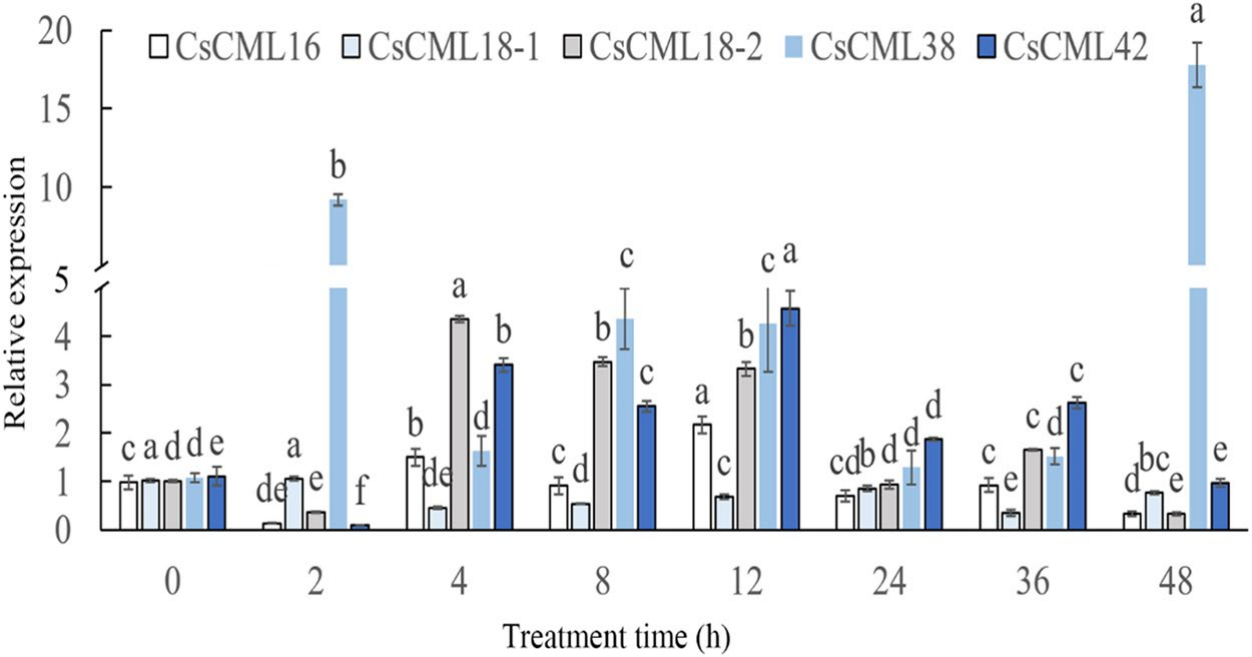
Expression profiles of *CsCML* genes in response to ABA treatment. Group comparison was performed with 0 h as control. Different small letters represent significant difference (*P* < 0.05).

ABA, an important plant hormone, plays vital roles in the regulation of plant adaptation to surrounding environment. With the induction of ABA treatment, the expression of *CsCML38* showed the highest change, indicating that *CsCML38* was very sensitive to ABA treatment. The expression level of *CsCML16* significantly increased and reached maximum at 12 h, followed by an acute decrease at 24 h. The expression level of *CsCML18-1* rapidly decreased at 4 h, and the induction of *CsCML18-1* steadily increased until 24 h. The expression level of *CsCML18-2* increased significantly at 4 h, then decreased gradually. The expression level of *CsCML42* declined at 2 h, followed by an increase at 12 h, and a decrease at 24 h (Fig. 6).

**Figure 7:**
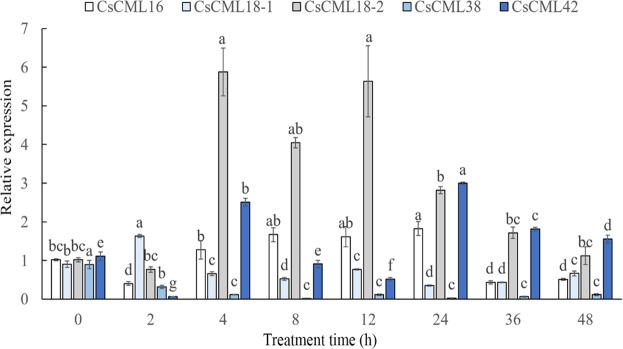
Expression profiles of *CsCML* genes in response to salt stress. Group comparison was performed with 0 h as control. Different small letters represent significant difference (*P* < 0.05).

Salt stress is a common threat which restricts growth and development of plants. After NaCl treatment, the expression levels of *CsCML16*, *CsCML18-2* and *CsCML42* were increased significantly with the highest expression levels during 4–24 h after stress. However, the expression of *CsCML38* decreased after salt stress, indicating that salt stress could suppress the expression of *CsCML38*. The expression patterns of *CsCML16*, *CsCML18-2* and *CsCML42* under salt stress were similar to that under cold stress, suggesting that these genes may be involved in similar signal pathway in response to salt and cold stresses (Fig. 7).

## Discussion

Ca^2+^ ion is a critical second messenger and could induce various physiological responses in plants in response to diverse stimuli. CML, as a Ca^2+^ sensor, mediates interpretation of Ca^2+^ signals in plant cell and also plays an important role in plants respond to various environmental stresses. In the present study, five *CML* genes namely *CsCML16*, *CsCML18-1*, *CsCML18-2*, *CsCML38* and *CsCML42* were identified and cloned from tea plant leaves. These genes encode small proteins containing 136–201 amino acids. Apart from CML18-1 protein which contains only one EF-hand motif, four proteins were found to have two EF-hand motifs. The *CsCML* gene family in tea plants contains EF-hand motifs with no other identifiable functional domains. These findings are in accordance with previously reports in *Arabidopsis*, rice and tomato^10,17,24^. Phylogenetic analysis revealed that amino acid sequences of putative *Cs*CMLs from tea plant have high similarity with CMLs from other plant species. These results indicated that the close evolutionary relationship existed in plant CML proteins.

Furthermore, the expression profiles of *CsCML* in different tissues indicated that *CsCML* genes were differentially expressed in tea plant. Expression levels of *CsCML16* and *CsCML18-1* in flowers were significantly higher than in other tissues. Interestingly, the expression levels of *CsCML16* and *CsCML18* were lower in flowers of *Arabidopsi*s^24^, whereas *AtCML24* and *AtCML25* strongly affected the pollen germination and and pollen tube growth^25,26^. It can; therefore, be suggested that *CsCML16* and *CsCML18-1* expressed differently to adapt different species. Conversely, expression levels of *CsCML18-2*, *CsCML38* and *CsCML42* in roots were significantly higher than in leaves, stem and flower organs, suggesting that different *CML* gene members from different species have distinct expression levels in various tissues, and may function in different physiological processes. The diversified expression of these *CsCML* genes revealed that they might play significant role at different plant developmental stages.

It is known that *CML* genes are involved in response to different environmental stresses and hormones. ABA plays an important role in cellular signal transduction in response to abiotic stresses. It could regulate the expression of several ABA-responsive genes through cellular Ca^2+^ ion changes^27^. Over expression of *Oryza sativa* multi-stress-responsive gene 2 (*OsMSR2*), which was a novel *CML* gene, improved the drought and salt tolerance of plants via ABA-mediated pathways^28^. Subsequently, another novel *CML* gene named *OsDSR1* (*Oryza sativa drought stress response-1*) showed greater sensitivity to ABA during the development of plant^29^. *OsCML4* could improve drought tolerance by effectively scavenging of ROS in rice^17^. In Arabidopsis, some studies have reported that *AtCML24* was induced by cold and drought stress, suggest that *AtCML24* might be involved in cold-related Ca^2+^ signals transduction^30^. *AtCML37*, *AtCML39* and *AtCML38* were also in response to several stimulus-induced and developmental signalling pathways, including salt, drought, and ABA^31^, while *AtCML42* may function to increase resistance to pathogens^32^. *MtCML40* in *Medicago truncatula* was reported to be involved in salt, cold tolerance as well as ABA treatment^33^. Altogether, accumulating evidence has demonstrated that CML proteins play important roles in Ca^2+^ signal transduction during plant growth and adaptation to abiotic stress.

The transcriptional regulation analysis under abiotic stress and hormones showed that the expression level of *CsCMLs* was affected in tea plants. The results revealed that the expression levels of *CsCML16*, *CsCML18-2* and *CsCML42* were induced significantly under low temperature condition, suggesting that these genes were involved in cold tolerance. Previous investigations have confirmed that *CMLs* are involved in plant responses to cold stress. For example, cold treatment could induce the expression levels of *AtCML24* and *OsMSR2*, which might participate in cold-induced Ca^2+^ signal transduction^28,30^.

Under drought stress, the expression levels of *CsCML16* and *CsCML18-1* decreased. This result is in contrast with the study by Jung *et al*.^34^ who reported that *OsCML16* was involved in drought tolerance through enhanced root growth. *CML38* is a likely homolog of an endogenous suppressor of antiviral silencing^35,36^. In the present study, *CsCML38* was responsive to all treatments except cold stress. However, the molecular mechanism of *CsCML38* in response to abiotic stresses remains unclear. Whether it is similar to other *CsCML* homologs needs to be verified in the future study. The result reveals that *CsCML* genes have diverse functions in response to different stimuli. Meanwhile, the role of *CsCML* genes may be activated by different signals.

Both *CsCML18-2* and *CsCML42* were up-regulated under low temperature and ABA treatments. This result indicates that *CMLs* are links of signal pathways which involved in different stimuli. It is in accordance with the role of a CDPK in ABA-dependent cold acclimation^37^. The up-regulated *CsCML16*, *CsCML18-2* and *CsCML42* in the present study suggest that these genes are involved in high salinity tolerance. The function of these salt responsive *CML* genes in the regulation of salt tolerance may be due to targeting high affinity K^+^ transporter-dependent Na^+^ accumulation^38^.

In summary, our results indicated that *CsCMLs* possibly function as stress-responsive genes to improve stress tolerance in tea plant. Although the study of *CsCML* family genes is still largely unexplored; our results, to some extent, advanced the understanding of the biological function of *CsCML* genes in tea plant and supply theory foundation for breeding stress-resistant tea cultivars.

## Materials and Methods

### Plant materials and stress treatment

Two-year-old tea plant seedlings (*Camellia sinensis* cv. Longjing Changye) were used in this study. The tea seedlings were selected and cultured in a growth chamber maintained at 24/22 °C day/night temperature with 75 ± 5% humidity and a photoperiod of 12 h light (200 μmol m^−2^ s^−1^) and 12 h dark. To evaluate the expression patterns of *CsCML* genes in different tissues, the leaves, roots, old stems, immature stems and flowers from the tea plant were collected. To determine the expression changes of the *CsCML* genes under various abiotic stresses, time-course experiments were carried out. After one week, the plants were treated with low temperature (10 °C), drought [PEG-6000, 20% (w/v)], abscisic acid (ABA, 200 mg/L) and NaCl (12 g/L), respectively. The third leaves from the top of tea plant were picked at 0, 2, 4, 8, 12, 24, 36 and 48 h after treatments. The time point of 0 h was set as control. The picked samples were frozen in liquid nitrogen immediately and stored at −80 °C for RNA extraction. Each sample has three biological replicates.

### RNA extraction and cDNA synthesis

Total RNA was isolated using EASYspin Plant RNA Kit (Aidlab, Beijing, China) following manufacturer’s instruction. RNA quality was checked on Agilent 2100 Bioanalyzer (Agilent, USA). First strand cDNA was produced using Revert Aid™ First Strand cDNA Synthesis Kit (Thermo, America) according to manufacturer’s instruction, and stored at −20 °C.

### Amplification and cloning of *CsCML* genes from tea plant

In order to find the *CsCML* family genes of tea plants, *CsCML21* nucleic acid sequence (GenBank accession: JQ999983) and the transcriptome data (SRA accession number: SRR5075641) were used for homogenous alignment by using BLAST program. The ORF regions of *CsCML* genes were generated by RT-PCR using primers in Table 2. PCR amplification was conducted by the following program: 94 °C for 2 min, 35 cycles of amplification at 94 °C for 30 s, 55 °C for 30 s, 72 °C for 2 min, followed by final extension at 72 °C for 10 min. The PCR products were loaded on 1.2% agarose gels for electrophoretic analysis and purified by a DNA purification kit (Qiagen, Valencia, CA). The purified DNA products was ligated into TOPO-TA vector and transformed into component cells (*E. coli* DH5α). Three independent clones from each isolate were sequenced by GenScript company (Nanjing, China).

**Table 2:** Primers used for the amplification of ORF regions of *CsCML* genes.

Name	Primer sequences 5′-3′
*CsCML16*	F: ATGACTATGCTTCAATCCGA R: CACGGTGAGGCCAAGAAAAT
*CsCML 18-1*	F: ATGGCGAAGAGTTCAAGCAA R: TCAAGCCTTCATCATCTTCT
*CsCML 18-2*	F: ATGGCCAATAGTCCGAGTAA R: TCAAAGCCTCATCATCTTCT
*CsCML 38*	F: ATGGAGAAGAGCTTAAGCCA R: CTAAGACATCATAATCCTAA
*CsCML 42*	F: ATGGAAACTACTGCTGGAACTC R: TGATTAAGCGCTGCGAACCA

### Nucleotide and amino acid sequences analysis

Phylogenetic analysis of the *CML* gene sequences was carried out by using BLAST program in the National Center for Biotechnology Information website (http://www.ncbi.nlm.nih.gov/BLAST). The phylogenetic tree was carried out by MEGA 7.0 using the neighbor-joining method. The predictions of possible open reading frame (ORF), theoretical isoelectric point (pI), and the molecular mass (Mw) were performed using online software (https://web.expasy.org/protparam/). Multiple sequence alignments of selected amino acids were conducted using ClustalW software. The MEME Suite software (http://meme-suite.org/tools/meme) was used for identifying sequence of EF-hand motif. The SWISS MODEL server (http://swissmodel.expasy.org/interactive) was used to predict and generate 3D structures of the CML members.

### Quantitative real-time PCR (qRT-PCR) analysis

qRT-PCR was performed to investigate the expression profiles of *CsCML* genes in different tissues and treatments. The cDNA from all tissues and treatments were used as template. The *GAPDH* (Accession No. GE651107) of tea plants was selected as the reference gene. The primer pairs are listed in Table 3. The qRT-PCR was conducted on a LightCycler^®^480 software (Roche) using FastStart Universal SYBR Green Master (Rox, Shanghai, China) in a 25 μL reaction mixture, containing 2 μL sample cDNA, 12.5 μL SYBR Green Master mixture, 0.5 μL each specific primer and 9.5 μL nuclease-free water. The qRT-PCR program included 95 °C for 5 min followed by 40 cycles at 95 °C for 10 s, 55 °C for 20 s and 72 °C for 30 s. At the end of experiment, the relative gene expression levels were calculated using the 2^−ΔΔCT^ method^39^.

**Table 3:** Primers used for the qRT-PCR analysis in this study.

Name	Primer sequences 5′-3′
*CsCML16*	F: ATGACTATGCTTCAATCCGA R: CACGGTGAGGCCAAGAAAAT
*CsCML18-1*	F: GAAGTCCAGCGAGTAATGTC R: TCAAGCCTTCATCATCTTCT
*CsCML18-2*	F: AAGAGATTGGGAGAGAAGTG R: TCAAAGCCTCATCATCTTCT
*CsCML38*	F: GGCGATGGAAATGGCAAGAT R: ATTGAGAACACCATCGCCAT
*CsCML42*	F: GTCTCAACTCCCTCCGTCTC R: TATCCATCGCCGTCCTCATC
*GAPDH*	F: TTGGCATCGTTGAGGGTCT R: CAGTGGGAACACGGAAAGC

### Statistical analysis

The statistical analyses were conducted by Microsoft EXCEL 2016 and SPSS 22.0 (https://www.ibm.com/products/spss-statistics). Difference significance analysis was performed using One-way ANOVA analysis and *P* < 0.05 was considered as significant difference.
